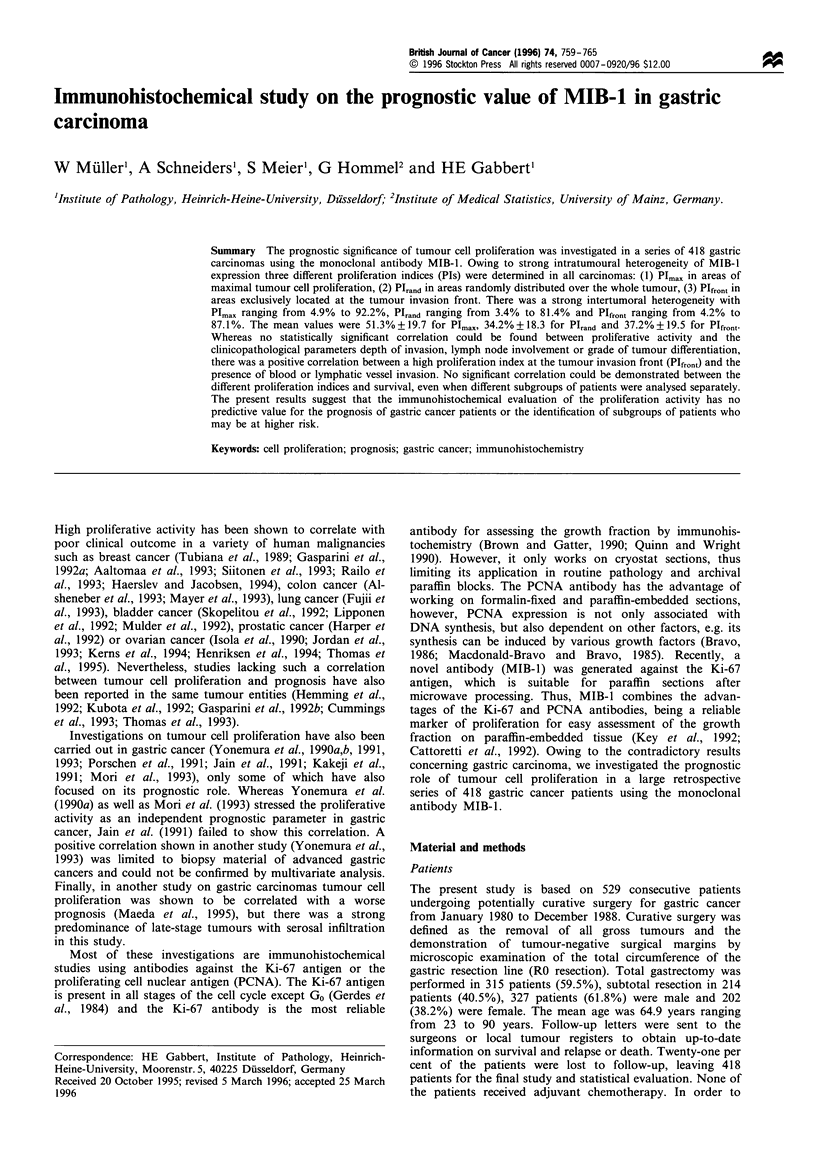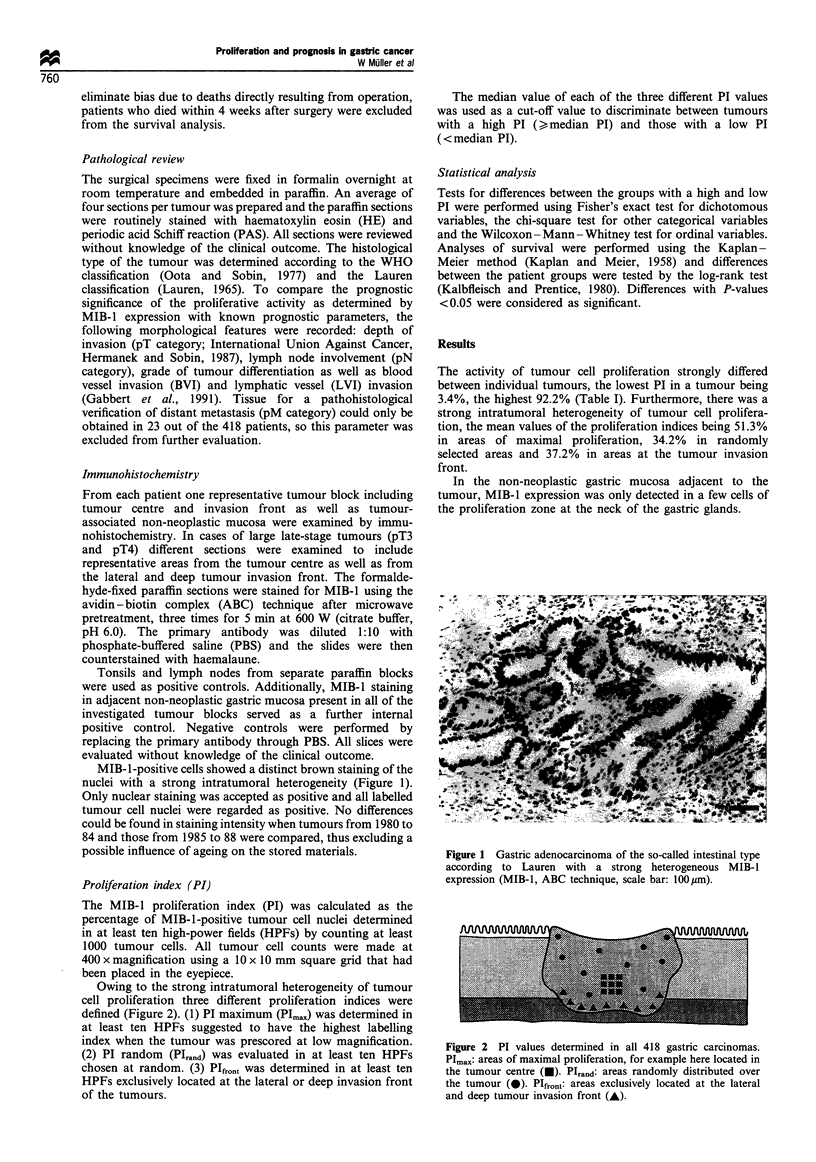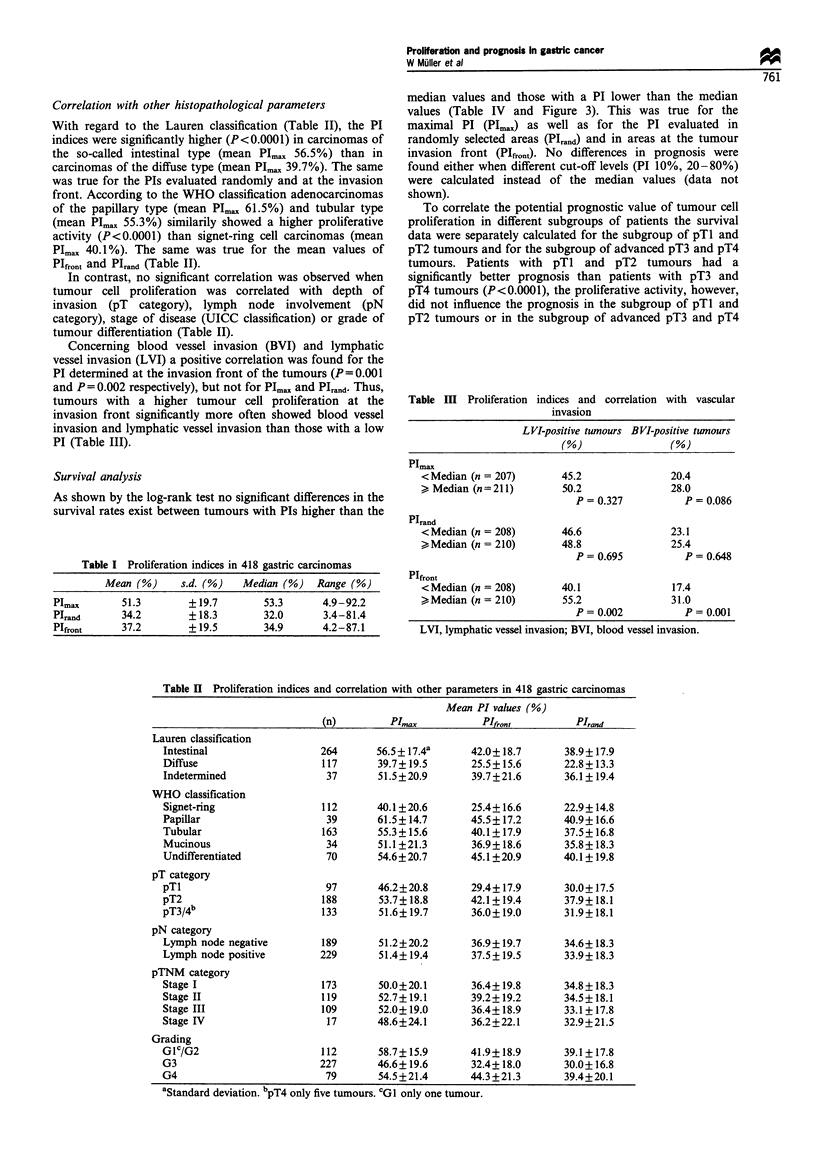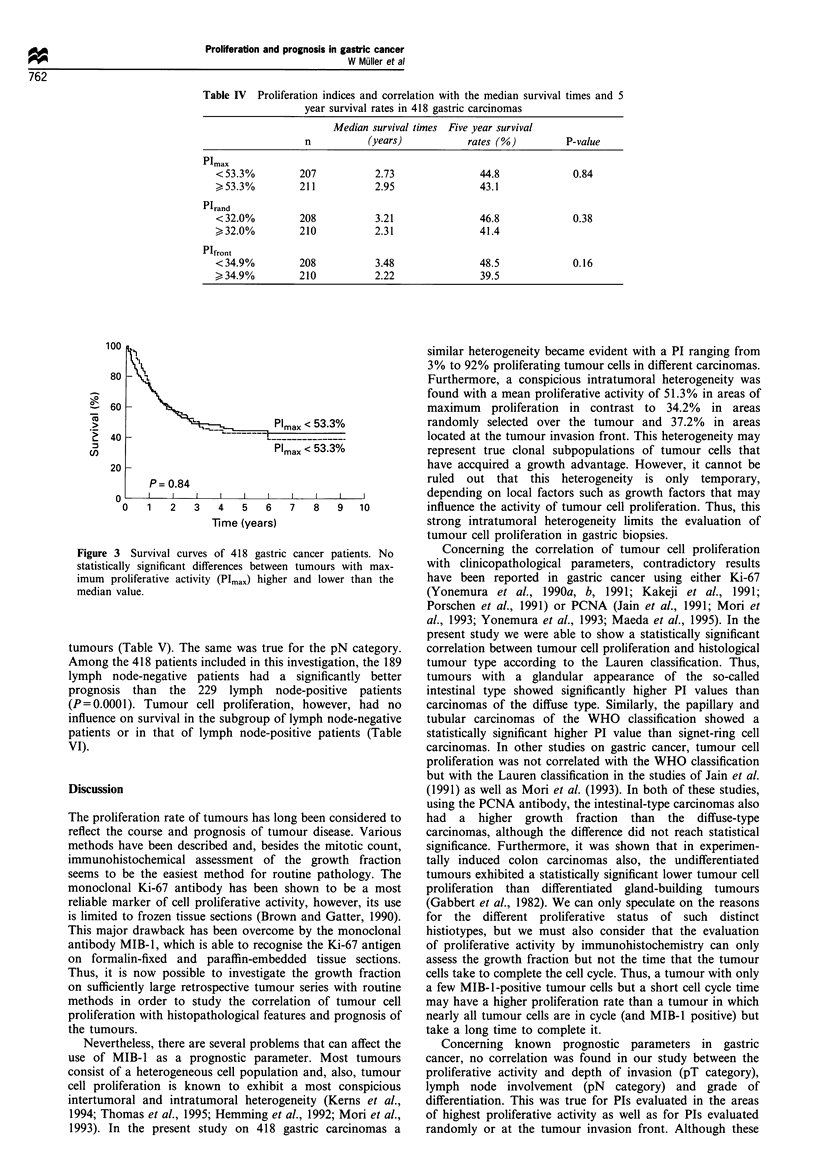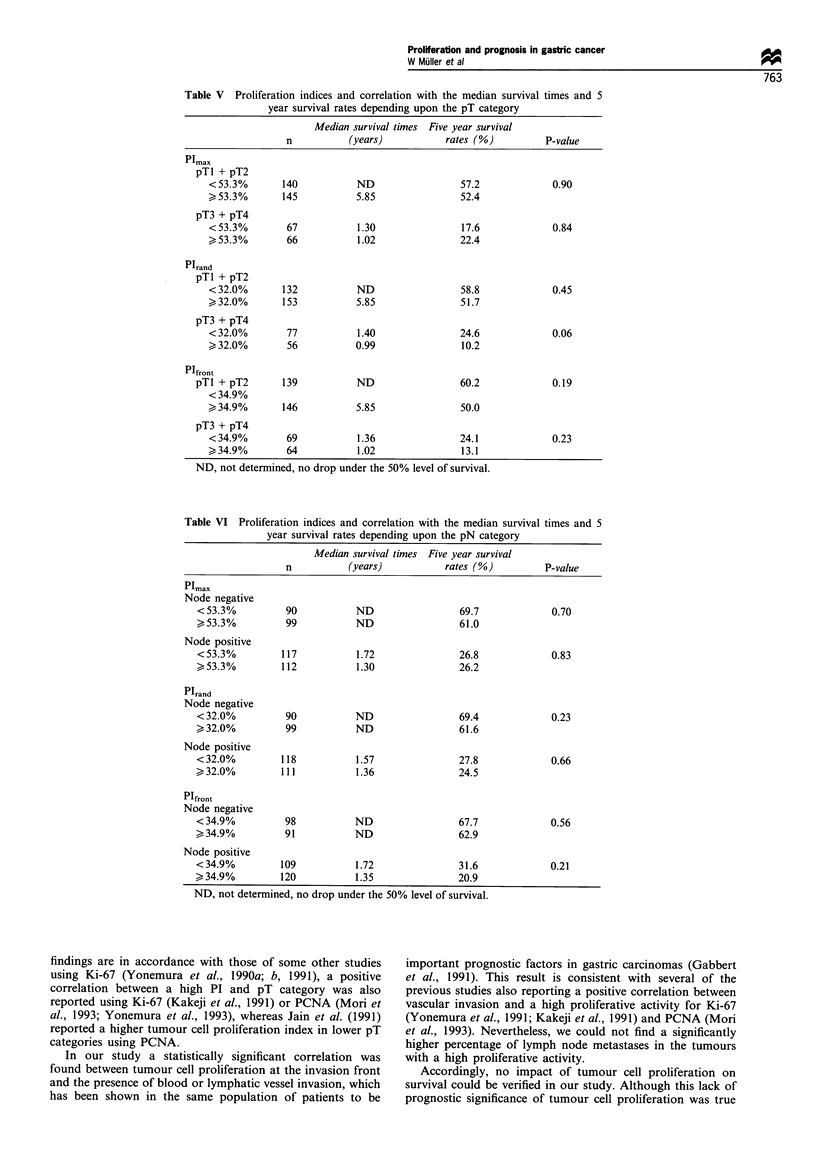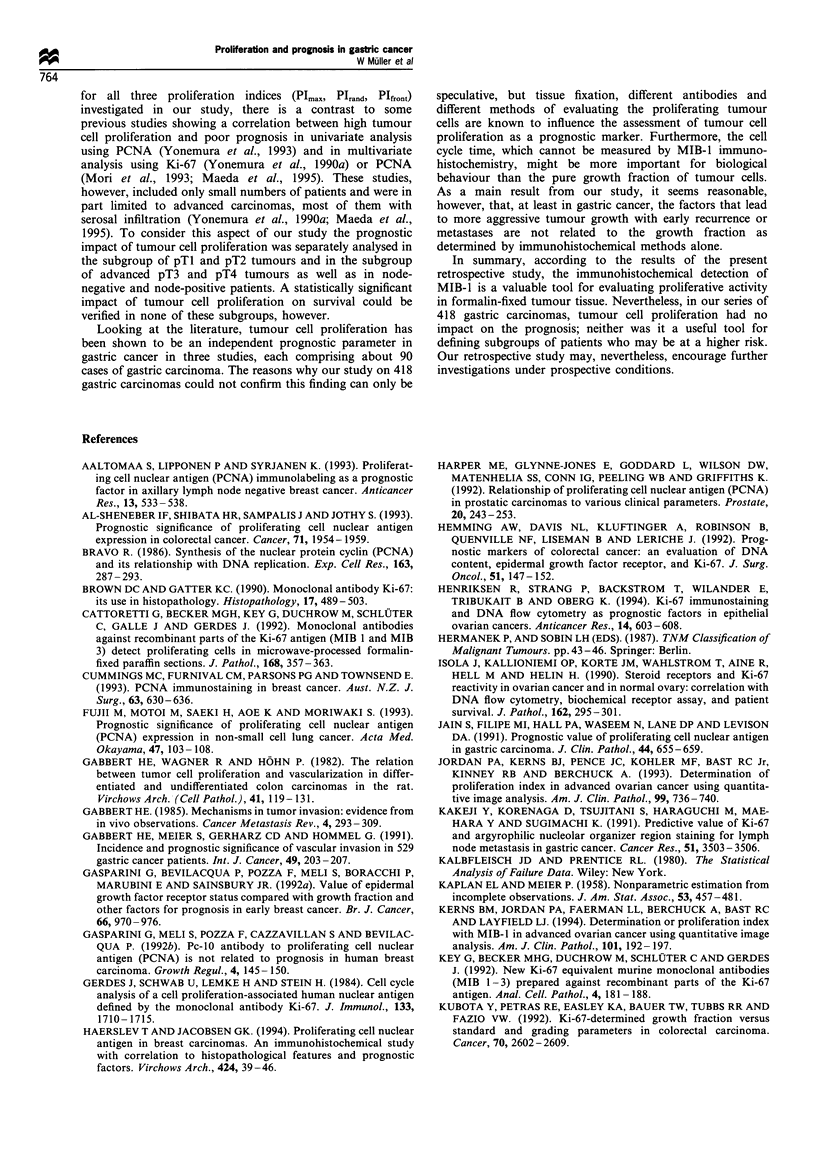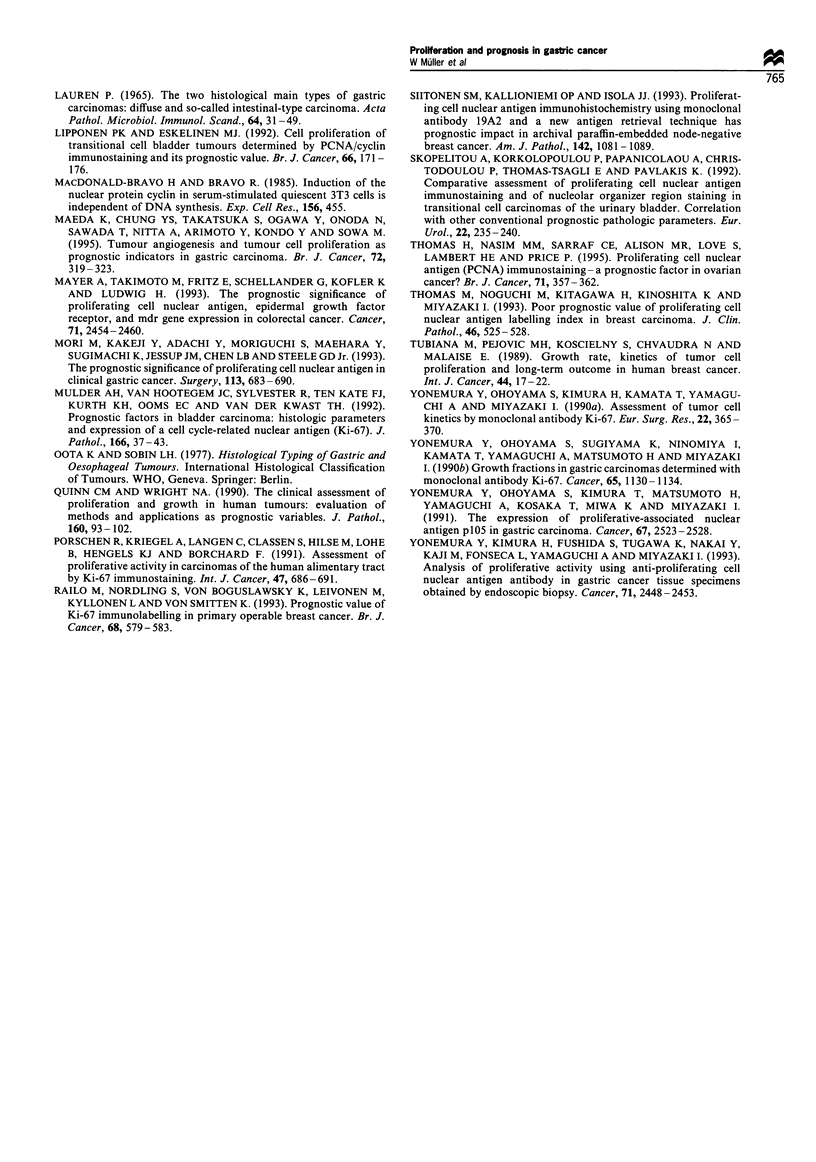# Immunohistochemical study on the prognostic value of MIB-1 in gastric carcinoma.

**DOI:** 10.1038/bjc.1996.433

**Published:** 1996-09

**Authors:** W. Müller, A. Schneiders, S. Meier, G. Hommel, H. E. Gabbert

**Affiliations:** Institute of Pathology, Heinrich-Heine-University, University of Mainz, Germany.

## Abstract

**Images:**


British Journal of Cancer (1996) 74, 759-765
© 1996 Stockton Press All rights reserved 0007-0920/96 $12.00

### Immunohistochemical study on the prognostic value of MIB-1 in gastric carcinoma

W Müller[1], A Schneiders[1], S Meier[1], G Hommel[2] and HE Gabbert[1]

[1]*Institute of Pathology, Heinrich-Heine-University, Düsseldorf;* [2]*Institute of Medical Statistics, University of Mainz, Germany.*

**Summary** The prognostic significance of tumour cell proliferation was investigated in a series of 418 gastric carcinomas using the monoclonal antibody MIB-1. Owing to strong intratumoural heterogeneity of MIB-1 expression three different proliferation indices (PIs) were determined in all carcinomas: (1) $PI_{max}$ in areas of maximal tumour cell proliferation, (2) $PI_{rand}$ in areas randomly distributed over the whole tumour, (3) $PI_{front}$ in areas exclusively located at the tumour invasion front. There was a strong intertumoral heterogeneity with $PI_{max}$ ranging from 4.9% to 92.2%, $PI_{rand}$ ranging from 3.4% to 81.4% and $PI_{front}$ ranging from 4.2% to 87.1%. The mean values were 51.3% ± 19.7 for $PI_{max}$, 34.2% ± 18.3 for $PI_{rand}$ and 37.2% ± 19.5 for $PI_{front}$. Whereas no statistically significant correlation could be found between proliferative activity and the clinicopathological parameters depth of invasion, lymph node involvement or grade of tumour differentiation, there was a positive correlation between a high proliferation index at the tumour invasion front ($PI_{front}$) and the presence of blood or lymphatic vessel invasion. No significant correlation could be demonstrated between the different proliferation indices and survival, even when different subgroups of patients were analysed separately. The present results suggest that the immunohistochemical evaluation of the proliferation activity has no predictive value for the prognosis of gastric cancer patients or the identification of subgroups of patients who may be at higher risk.

**Keywords:** cell proliferation; prognosis; gastric cancer; immunohistochemistry

High proliferative activity has been shown to correlate with poor clinical outcome in a variety of human malignancies such as breast cancer (Tubiana *et al.*, 1989; Gasparini *et al.*, 1992*a*; Aaltomaa *et al.*, 1993; Siitonen *et al.*, 1993; Railo *et al.*, 1993; Haerslev and Jacobsen, 1994), colon cancer (Al-sheneber *et al.*, 1993; Mayer *et al.*, 1993), lung cancer (Fujii *et al.*, 1993), bladder cancer (Skopelitou *et al.*, 1992; Lipponen *et al.*, 1992; Mulder *et al.*, 1992), prostatic cancer (Harper *et al.*, 1992) or ovarian cancer (Isola *et al.*, 1990; Jordan *et al.*, 1993; Kerns *et al.*, 1994; Henriksen *et al.*, 1994; Thomas *et al.*, 1995). Nevertheless, studies lacking such a correlation between tumour cell proliferation and prognosis have also been reported in the same tumour entities (Hemming *et al.*, 1992; Kubota *et al.*, 1992; Gasparini *et al.*, 1992*b*; Cummings *et al.*, 1993; Thomas *et al.*, 1993).

Investigations on tumour cell proliferation have also been carried out in gastric cancer (Yonemura *et al.*, 1990*a,b*, 1991, 1993; Porschen *et al.*, 1991; Jain *et al.*, 1991; Kakeji *et al.*, 1991; Mori *et al.*, 1993), only some of which have also focused on its prognostic role. Whereas Yonemura *et al.* (1990*a*) as well as Mori *et al.* (1993) stressed the proliferative activity as an independent prognostic parameter in gastric cancer, Jain *et al.* (1991) failed to show this correlation. A positive correlation shown in another study (Yonemura *et al.*, 1993) was limited to biopsy material of advanced gastric cancers and could not be confirmed by multivariate analysis. Finally, in another study on gastric carcinomas tumour cell proliferation was shown to be correlated with a worse prognosis (Maeda *et al.*, 1995), but there was a strong predominance of late-stage tumours with serosal infiltration in this study.

Most of these investigations are immunohistochemical studies using antibodies against the Ki-67 antigen or the proliferating cell nuclear antigen (PCNA). The Ki-67 antigen is present in all stages of the cell cycle except $G_0$ (Gerdes *et al.*, 1984) and the Ki-67 antibody is the most reliable antibody for assessing the growth fraction by immunohistochemistry (Brown and Gatter, 1990; Quinn and Wright 1990). However, it only works on cryostat sections, thus limiting its application in routine pathology and archival paraffin blocks. The PCNA antibody has the advantage of working on formalin-fixed and paraffin-embedded sections, however, PCNA expression is not only associated with DNA synthesis, but also dependent on other factors, e.g. its synthesis can be induced by various growth factors (Bravo, 1986; Macdonald-Bravo and Bravo, 1985). Recently, a novel antibody (MIB-1) was generated against the Ki-67 antigen, which is suitable for paraffin sections after microwave processing. Thus, MIB-1 combines the advantages of the Ki-67 and PCNA antibodies, being a reliable marker of proliferation for easy assessment of the growth fraction on paraffin-embedded tissue (Key *et al.*, 1992; Cattoretti *et al.*, 1992). Owing to the contradictory results concerning gastric carcinoma, we investigated the prognostic role of tumour cell proliferation in a large retrospective series of 418 gastric cancer patients using the monoclonal antibody MIB-1.

### Material and methods

### *Patients*

The present study is based on 529 consecutive patients undergoing potentially curative surgery for gastric cancer from January 1980 to December 1988. Curative surgery was defined as the removal of all gross tumours and the demonstration of tumour-negative surgical margins by microscopic examination of the total circumference of the gastric resection line (R0 resection). Total gastrectomy was performed in 315 patients (59.5%), subtotal resection in 214 patients (40.5%), 327 patients (61.8%) were male and 202 (38.2%) were female. The mean age was 64.9 years ranging from 23 to 90 years. Follow-up letters were sent to the surgeons or local tumour registers to obtain up-to-date information on survival and relapse or death. Twenty-one per cent of the patients were lost to follow-up, leaving 418 patients for the final study and statistical evaluation. None of the patients received adjuvant chemotherapy. In order to

Correspondence: HE Gabbert, Institute of Pathology, Heinrich-Heine-University, Moorenstr. 5, 40225 Düsseldorf, Germany
Received 20 October 1995; revised 5 March 1996; accepted 25 March 1996

eliminate bias due to deaths directly resulting from operation, patients who died within 4 weeks after surgery were excluded from the survival analysis.

*Pathological review*

The surgical specimens were fixed in formalin overnight at room temperature and embedded in paraffin. An average of four sections per tumour was prepared and the paraffin sections were routinely stained with haematoxylin eosin (HE) and periodic acid Schiff reaction (PAS). All sections were reviewed without knowledge of the clinical outcome. The histological type of the tumour was determined according to the WHO classification (Oota and Sobin, 1977) and the Lauren classification (Lauren, 1965). To compare the prognostic significance of the proliferative activity as determined by MIB-1 expression with known prognostic parameters, the following morphological features were recorded: depth of invasion (pT category; International Union Against Cancer, Hermanek and Sobin, 1987), lymph node involvement (pN category), grade of tumour differentiation as well as blood vessel invasion (BVI) and lymphatic vessel (LVI) invasion (Gabbert *et al.*, 1991). Tissue for a pathohistological verification of distant metastasis (pM category) could only be obtained in 23 out of the 418 patients, so this parameter was excluded from further evaluation.

*Immunohistochemistry*

From each patient one representative tumour block including tumour centre and invasion front as well as tumour-associated non-neoplastic mucosa were examined by immunohistochemistry. In cases of large late-stage tumours (pT3 and pT4) different sections were examined to include representative areas from the tumour centre as well as from the lateral and deep tumour invasion front. The formaldehyde-fixed paraffin sections were stained for MIB-1 using the avidin–biotin complex (ABC) technique after microwave pretreatment, three times for 5 min at 600 W (citrate buffer, pH 6.0). The primary antibody was diluted 1:10 with phosphate-buffered saline (PBS) and the slides were then counterstained with haemalaune.

Tonsils and lymph nodes from separate paraffin blocks were used as positive controls. Additionally, MIB-1 staining in adjacent non-neoplastic gastric mucosa present in all of the investigated tumour blocks served as a further internal positive control. Negative controls were performed by replacing the primary antibody through PBS. All slices were evaluated without knowledge of the clinical outcome.

MIB-1-positive cells showed a distinct brown staining of the nuclei with a strong intratumoral heterogeneity (Figure 1). Only nuclear staining was accepted as positive and all labelled tumour cell nuclei were regarded as positive. No differences could be found in staining intensity when tumours from 1980 to 84 and those from 1985 to 88 were compared, thus excluding a possible influence of ageing on the stored materials.

*Proliferation index (PI)*

The MIB-1 proliferation index (PI) was calculated as the percentage of MIB-1-positive tumour cell nuclei determined in at least ten high-power fields (HPFs) by counting at least 1000 tumour cells. All tumour cell counts were made at 400 × magnification using a 10 × 10 mm square grid that had been placed in the eyepiece.

Owing to the strong intratumoral heterogeneity of tumour cell proliferation three different proliferation indices were defined (Figure 2). (1) PI maximum ($PI_{max}$) was determined in at least ten HPFs suggested to have the highest labelling index when the tumour was prescored at low magnification. (2) PI random ($PI_{rand}$) was evaluated in at least ten HPFs chosen at random. (3) $PI_{front}$ was determined in at least ten HPFs exclusively located at the lateral or deep invasion front of the tumours.

The median value of each of the three different PI values was used as a cut-off value to discriminate between tumours with a high PI (⩾median PI) and those with a low PI (<median PI).

*Statistical analysis*

Tests for differences between the groups with a high and low PI were performed using Fisher's exact test for dichotomous variables, the chi-square test for other categorical variables and the Wilcoxon–Mann–Whitney test for ordinal variables. Analyses of survival were performed using the Kaplan–Meier method (Kaplan and Meier, 1958) and differences between the patient groups were tested by the log-rank test (Kalbfleisch and Prentice, 1980). Differences with *P*-values <0.05 were considered as significant.

### Results

The activity of tumour cell proliferation strongly differed between individual tumours, the lowest PI in a tumour being 3.4%, the highest 92.2% (Table I). Furthermore, there was a strong intratumoral heterogeneity of tumour cell proliferation, the mean values of the proliferation indices being 51.3% in areas of maximal proliferation, 34.2% in randomly selected areas and 37.2% in areas at the tumour invasion front.

In the non-neoplastic gastric mucosa adjacent to the tumour, MIB-1 expression was only detected in a few cells of the proliferation zone at the neck of the gastric glands.

**Figure 1** Gastric adenocarcinoma of the so-called intestinal type according to Lauren with a strong heterogeneous MIB-1 expression (MIB-1, ABC technique, scale bar: 100 μm).

**Figure 2** PI values determined in all 418 gastric carcinomas. $PI_{max}$: areas of maximal proliferation, for example here located in the tumour centre (■). $PI_{rand}$: areas randomly distributed over the tumour (●). $PI_{front}$: areas exclusively located at the lateral and deep tumour invasion front (▲).

*Correlation with other histopathological parameters*

With regard to the Lauren classification (Table II), the PI indices were significantly higher ($P<0.0001$) in carcinomas of the so-called intestinal type (mean $PI_{max}$ 56.5%) than in carcinomas of the diffuse type (mean $PI_{max}$ 39.7%). The same was true for the PIs evaluated randomly and at the invasion front. According to the WHO classification adenocarcinomas of the papillary type (mean $PI_{max}$ 61.5%) and tubular type (mean $PI_{max}$ 55.3%) similarily showed a higher proliferative activity ($P<0.0001$) than signet-ring cell carcinomas (mean $PI_{max}$ 40.1%). The same was true for the mean values of $PI_{front}$ and $PI_{rand}$ (Table II).

In contrast, no significant correlation was observed when tumour cell proliferation was correlated with depth of invasion (pT category), lymph node involvement (pN category), stage of disease (UICC classification) or grade of tumour differentiation (Table II).

Concerning blood vessel invasion (BVI) and lymphatic vessel invasion (LVI) a positive correlation was found for the PI determined at the invasion front of the tumours ($P=0.001$ and $P=0.002$ respectively), but not for $PI_{max}$ and $PI_{rand}$. Thus, tumours with a higher tumour cell proliferation at the invasion front significantly more often showed blood vessel invasion and lymphatic vessel invasion than those with a low PI (Table III).

*Survival analysis*

As shown by the log-rank test no significant differences in the survival rates exist between tumours with PIs higher than the median values and those with a PI lower than the median values (Table IV and Figure 3). This was true for the maximal PI ($PI_{max}$) as well as for the PI evaluated in randomly selected areas ($PI_{rand}$) and in areas at the tumour invasion front ($PI_{front}$). No differences in prognosis were found either when different cut-off levels (PI 10%, 20–80%) were calculated instead of the median values (data not shown).

To correlate the potential prognostic value of tumour cell proliferation in different subgroups of patients the survival data were separately calculated for the subgroup of pT1 and pT2 tumours and for the subgroup of advanced pT3 and pT4 tumours. Patients with pT1 and pT2 tumours had a significantly better prognosis than patients with pT3 and pT4 tumours ($P<0.0001$), the proliferative activity, however, did not influence the prognosis in the subgroup of pT1 and pT2 tumours or in the subgroup of advanced pT3 and pT4

**Table I** Proliferation indices in 418 gastric carcinomas

| | *Mean (%)* | *s.d. (%)* | *Median (%)* | *Range (%)* |
|---|---|---|---|---|
| $PI_{max}$ | 51.3 | ±19.7 | 53.3 | 4.9–92.2 |
| $PI_{rand}$ | 34.2 | ±18.3 | 32.0 | 3.4–81.4 |
| $PI_{front}$ | 37.2 | ±19.5 | 34.9 | 4.2–87.1 |

**Table III** Proliferation indices and correlation with vascular invasion

| | *LVI-positive tumours (%)* | *BVI-positive tumours (%)* |
|---|---|---|
| $PI_{max}$ | | |
| <Median ($n$ = 207) | 45.2 | 20.4 |
| ⩾ Median ($n$=211) | 50.2 | 28.0 |
| | $P$ = 0.327 | $P$ = 0.086 |
| $PI_{rand}$ | | |
| <Median ($n$ = 208) | 46.6 | 23.1 |
| ⩾Median ($n$ = 210) | 48.8 | 25.4 |
| | $P$ = 0.695 | $P$ = 0.648 |
| $PI_{front}$ | | |
| <Median ($n$ = 208) | 40.1 | 17.4 |
| ⩾Median ($n$ = 210) | 55.2 | 31.0 |
| | $P$ = 0.002 | $P$ = 0.001 |

LVI, lymphatic vessel invasion; BVI, blood vessel invasion.

**Table II** Proliferation indices and correlation with other parameters in 418 gastric carcinomas

| | (n) | *Mean PI values (%)* $PI_{max}$ | $PI_{front}$ | $PI_{rand}$ |
|---|---|---|---|---|
| Lauren classification | | | | |
| Intestinal | 264 | 56.5±17.4[a] | 42.0±18.7 | 38.9±17.9 |
| Diffuse | 117 | 39.7±19.5 | 25.5±15.6 | 22.8±13.3 |
| Indetermined | 37 | 51.5±20.9 | 39.7±21.6 | 36.1±19.4 |
| WHO classification | | | | |
| Signet-ring | 112 | 40.1±20.6 | 25.4±16.6 | 22.9±14.8 |
| Papillar | 39 | 61.5±14.7 | 45.5±17.2 | 40.9±16.6 |
| Tubular | 163 | 55.3±15.6 | 40.1±17.9 | 37.5±16.8 |
| Mucinous | 34 | 51.1±21.3 | 36.9±18.6 | 35.8±18.3 |
| Undifferentiated | 70 | 54.6±20.7 | 45.1±20.9 | 40.1±19.8 |
| pT category | | | | |
| pT1 | 97 | 46.2±20.8 | 29.4±17.9 | 30.0±17.5 |
| pT2 | 188 | 53.7±18.8 | 42.1±19.4 | 37.9±18.1 |
| pT3/4[b] | 133 | 51.6±19.7 | 36.0±19.0 | 31.9±18.1 |
| pN category | | | | |
| Lymph node negative | 189 | 51.2±20.2 | 36.9±19.7 | 34.6±18.3 |
| Lymph node positive | 229 | 51.4±19.4 | 37.5±19.5 | 33.9±18.3 |
| pTNM category | | | | |
| Stage I | 173 | 50.0±20.1 | 36.4±19.8 | 34.8±18.3 |
| Stage II | 119 | 52.7±19.1 | 39.2±19.2 | 34.5±18.1 |
| Stage III | 109 | 52.0±19.0 | 36.4±18.9 | 33.1±17.8 |
| Stage IV | 17 | 48.6±24.1 | 36.2±22.1 | 32.9±21.5 |
| Grading | | | | |
| G1[c]/G2 | 112 | 58.7±15.9 | 41.9±18.9 | 39.1±17.8 |
| G3 | 227 | 46.6±19.6 | 32.4±18.0 | 30.0±16.8 |
| G4 | 79 | 54.5±21.4 | 44.3±21.3 | 39.4±20.1 |

[a]Standard deviation. [b]pT4 only five tumours. [c]G1 only one tumour.

**Table IV** Proliferation indices and correlation with the median survival times and 5 year survival rates in 418 gastric carcinomas

| | n | Median survival times (years) | Five year survival rates (%) | P-value |
|---|---|---|---|---|
| $PI_{max}$ | | | | |
| <53.3% | 207 | 2.73 | 44.8 | 0.84 |
| ⩾53.3% | 211 | 2.95 | 43.1 | |
| $PI_{rand}$ | | | | |
| <32.0% | 208 | 3.21 | 46.8 | 0.38 |
| ⩾32.0% | 210 | 2.31 | 41.4 | |
| $PI_{front}$ | | | | |
| <34.9% | 208 | 3.48 | 48.5 | 0.16 |
| ⩾34.9% | 210 | 2.22 | 39.5 | |

**Figure 3** Survival curves of 418 gastric cancer patients. No statistically significant differences between tumours with maximum proliferative activity ($PI_{max}$) higher and lower than the median value.

tumours (Table V). The same was true for the pN category. Among the 418 patients included in this investigation, the 189 lymph node-negative patients had a significantly better prognosis than the 229 lymph node-positive patients ($P = 0.0001$). Tumour cell proliferation, however, had no influence on survival in the subgroup of lymph node-negative patients or in that of lymph node-positive patients (Table VI).

### Discussion

The proliferation rate of tumours has long been considered to reflect the course and prognosis of tumour disease. Various methods have been described and, besides the mitotic count, immunohistochemical assessment of the growth fraction seems to be the easiest method for routine pathology. The monoclonal Ki-67 antibody has been shown to be a most reliable marker of cell proliferative activity, however, its use is limited to frozen tissue sections (Brown and Gatter, 1990). This major drawback has been overcome by the monoclonal antibody MIB-1, which is able to recognise the Ki-67 antigen on formalin-fixed and paraffin-embedded tissue sections. Thus, it is now possible to investigate the growth fraction on sufficiently large retrospective tumour series with routine methods in order to study the correlation of tumour cell proliferation with histopathological features and prognosis of the tumours.

Nevertheless, there are several problems that can affect the use of MIB-1 as a prognostic parameter. Most tumours consist of a heterogeneous cell population and, also, tumour cell proliferation is known to exhibit a most conspicious intertumoral and intratumoral heterogeneity (Kerns *et al.*, 1994; Thomas *et al.*, 1995; Hemming *et al.*, 1992; Mori *et al.*, 1993). In the present study on 418 gastric carcinomas a similar heterogeneity became evident with a PI ranging from 3% to 92% proliferating tumour cells in different carcinomas. Furthermore, a conspicious intratumoral heterogeneity was found with a mean proliferative activity of 51.3% in areas of maximum proliferation in contrast to 34.2% in areas randomly selected over the tumour and 37.2% in areas located at the tumour invasion front. This heterogeneity may represent true clonal subpopulations of tumour cells that have accquired a growth advantage. However, it cannot be ruled out that this heterogeneity is only temporary, depending on local factors such as growth factors that may influence the activity of tumour cell proliferation. Thus, this strong intratumoral heterogeneity limits the evaluation of tumour cell proliferation in gastric biopsies.

Concerning the correlation of tumour cell proliferation with clinicopathological parameters, contradictory results have been reported in gastric cancer using either Ki-67 (Yonemura *et al.*, 1990*a*, *b*, 1991; Kakeji *et al.*, 1991; Porschen *et al.*, 1991) or PCNA (Jain *et al.*, 1991; Mori *et al.*, 1993; Yonemura *et al.*, 1993; Maeda *et al.*, 1995). In the present study we were able to show a statistically significant correlation between tumour cell proliferation and histological tumour type according to the Lauren classification. Thus, tumours with a glandular appearance of the so-called intestinal type showed significantly higher PI values than carcinomas of the diffuse type. Similarly, the papillary and tubular carcinomas of the WHO classification showed a statistically significant higher PI value than signet-ring cell carcinomas. In other studies on gastric cancer, tumour cell proliferation was not correlated with the WHO classification but with the Lauren classification in the studies of Jain *et al.* (1991) as well as Mori *et al.* (1993). In both of these studies, using the PCNA antibody, the intestinal-type carcinomas also had a higher growth fraction than the diffuse-type carcinomas, although the difference did not reach statistical significance. Furthermore, it was shown that in experimentally induced colon carcinomas also, the undifferentiated tumours exhibited a statistically significant lower tumour cell proliferation than differentiated gland-building tumours (Gabbert *et al.*, 1982). We can only speculate on the reasons for the different proliferative status of such distinct histiotypes, but we must also consider that the evaluation of proliferative activity by immunohistochemistry can only assess the growth fraction but not the time that the tumour cells take to complete the cell cycle. Thus, a tumour with only a few MIB-1-positive tumour cells but a short cell cycle time may have a higher proliferation rate than a tumour in which nearly all tumour cells are in cycle (and MIB-1 positive) but take a long time to complete it.

Concerning known prognostic parameters in gastric cancer, no correlation was found in our study between the proliferative activity and depth of invasion (pT category), lymph node involvement (pN category) and grade of differentiation. This was true for PIs evaluated in the areas of highest proliferative activity as well as for PIs evaluated randomly or at the tumour invasion front. Although these

**Table V** Proliferation indices and correlation with the median survival times and 5 year survival rates depending upon the pT category

| | n | *Median survival times (years)* | *Five year survival rates (%)* | P-*value* |
|---|---|---|---|---|
| $PI_{max}$ | | | | |
| pT1 + pT2 | | | | |
| <53.3% | 140 | ND | 57.2 | 0.90 |
| ⩾53.3% | 145 | 5.85 | 52.4 | |
| pT3 + pT4 | | | | |
| <53.3% | 67 | 1.30 | 17.6 | 0.84 |
| ⩾53.3% | 66 | 1.02 | 22.4 | |
| $PI_{rand}$ | | | | |
| pT1 + pT2 | | | | |
| <32.0% | 132 | ND | 58.8 | 0.45 |
| ⩾32.0% | 153 | 5.85 | 51.7 | |
| pT3 + pT4 | | | | |
| <32.0% | 77 | 1.40 | 24.6 | 0.06 |
| ⩾32.0% | 56 | 0.99 | 10.2 | |
| $PI_{front}$ | | | | |
| pT1 + pT2 | 139 | ND | 60.2 | 0.19 |
| <34.9% | | | | |
| ⩾34.9% | 146 | 5.85 | 50.0 | |
| pT3 + pT4 | | | | |
| <34.9% | 69 | 1.36 | 24.1 | 0.23 |
| ⩾34.9% | 64 | 1.02 | 13.1 | |

ND, not determined, no drop under the 50% level of survival.

**Table VI** Proliferation indices and correlation with the median survival times and 5 year survival rates depending upon the pN category

| | n | *Median survival times (years)* | *Five year survival rates (%)* | P-*value* |
|---|---|---|---|---|
| $PI_{max}$ | | | | |
| Node negative | | | | |
| <53.3% | 90 | ND | 69.7 | 0.70 |
| ⩾53.3% | 99 | ND | 61.0 | |
| Node positive | | | | |
| <53.3% | 117 | 1.72 | 26.8 | 0.83 |
| ⩾53.3% | 112 | 1.30 | 26.2 | |
| $PI_{rand}$ | | | | |
| Node negative | | | | |
| <32.0% | 90 | ND | 69.4 | 0.23 |
| ⩾32.0% | 99 | ND | 61.6 | |
| Node positive | | | | |
| <32.0% | 118 | 1.57 | 27.8 | 0.66 |
| ⩾32.0% | 111 | 1.36 | 24.5 | |
| $PI_{front}$ | | | | |
| Node negative | | | | |
| <34.9% | 98 | ND | 67.7 | 0.56 |
| ⩾34.9% | 91 | ND | 62.9 | |
| Node positive | | | | |
| <34.9% | 109 | 1.72 | 31.6 | 0.21 |
| ⩾34.9% | 120 | 1.35 | 20.9 | |

ND, not determined, no drop under the 50% level of survival.

findings are in accordance with those of some other studies using Ki-67 (Yonemura *et al.*, 1990*a*; *b*, 1991), a positive correlation between a high PI and pT category was also reported using Ki-67 (Kakeji *et al.*, 1991) or PCNA (Mori *et al.*, 1993; Yonemura *et al.*, 1993), whereas Jain *et al.* (1991) reported a higher tumour cell proliferation index in lower pT categories using PCNA.

In our study a statistically significant correlation was found between tumour cell proliferation at the invasion front and the presence of blood or lymphatic vessel invasion, which has been shown in the same population of patients to be important prognostic factors in gastric carcinomas (Gabbert *et al.*, 1991). This result is consistent with several of the previous studies also reporting a positive correlation between vascular invasion and a high proliferative activity for Ki-67 (Yonemura *et al.*, 1991; Kakeji *et al.*, 1991) and PCNA (Mori *et al.*, 1993). Nevertheless, we could not find a significantly higher percentage of lymph node metastases in the tumours with a high proliferative activity.

Accordingly, no impact of tumour cell proliferation on survival could be verified in our study. Although this lack of prognostic significance of tumour cell proliferation was true for all three proliferation indices ($PI_{max}$, $PI_{rand}$, $PI_{front}$) investigated in our study, there is a contrast to some previous studies showing a correlation between high tumour cell proliferation and poor prognosis in univariate analysis using PCNA (Yonemura *et al.*, 1993) and in multivariate analysis using Ki-67 (Yonemura *et al.*, 1990*a*) or PCNA (Mori *et al.*, 1993; Maeda *et al.*, 1995). These studies, however, included only small numbers of patients and were in part limited to advanced carcinomas, most of them with serosal infiltration (Yonemura *et al.*, 1990*a*; Maeda *et al.*, 1995). To consider this aspect of our study the prognostic impact of tumour cell proliferation was separately analysed in the subgroup of pT1 and pT2 tumours and in the subgroup of advanced pT3 and pT4 tumours as well as in node-negative and node-positive patients. A statistically significant impact of tumour cell proliferation on survival could be verified in none of these subgroups, however.

Looking at the literature, tumour cell proliferation has been shown to be an independent prognostic parameter in gastric cancer in three studies, each comprising about 90 cases of gastric carcinoma. The reasons why our study on 418 gastric carcinomas could not confirm this finding can only be speculative, but tissue fixation, different antibodies and different methods of evaluating the proliferating tumour cells are known to influence the assessment of tumour cell proliferation as a prognostic marker. Furthermore, the cell cycle time, which cannot be measured by MIB-1 immunohistochemistry, might be more important for biological behaviour than the pure growth fraction of tumour cells. As a main result from our study, it seems reasonable, however, that, at least in gastric cancer, the factors that lead to more aggressive tumour growth with early recurrence or metastases are not related to the growth fraction as determined by immunohistochemical methods alone.

In summary, according to the results of the present retrospective study, the immunohistochemical detection of MIB-1 is a valuable tool for evaluating proliferative activity in formalin-fixed tumour tissue. Nevertheless, in our series of 418 gastric carcinomas, tumour cell proliferation had no impact on the prognosis; neither was it a useful tool for defining subgroups of patients who may be at a higher risk. Our retrospective study may, nevertheless, encourage further investigations under prospective conditions.

### References

AALTOMAA S, LIPPONEN P AND SYRJANEN K. (1993). Proliferating cell nuclear antigen (PCNA) immunolabeling as a prognostic factor in axillary lymph node negative breast cancer. *Anticancer Res.*, **13**, 533–538.

AL-SHENEBER IF, SHIBATA HR, SAMPALIS J AND JOTHY S. (1993). Prognostic significance of proliferating cell nuclear antigen expression in colorectal cancer. *Cancer*, **71**, 1954–1959.

BRAVO R. (1986). Synthesis of the nuclear protein cyclin (PCNA) and its relationship with DNA replication. *Exp. Cell Res.*, **163**, 287–293.

BROWN DC AND GATTER KC. (1990). Monoclonal antibody Ki-67: its use in histopathology. *Histopathology*, **17**, 489–503.

CATTORETTI G, BECKER MGH, KEY G, DUCHROW M, SCHLÜTER C, GALLE J AND GERDES J. (1992). Monoclonal antibodies against recombinant parts of the Ki-67 antigen (MIB 1 and MIB 3) detect proliferating cells in microwave-processed formalin-fixed paraffin sections. *J. Pathol.*, **168**, 357–363.

CUMMINGS MC, FURNIVAL CM, PARSONS PG AND TOWNSEND E. (1993). PCNA immunostaining in breast cancer. *Aust. N.Z. J. Surg.*, **63**, 630–636.

FUJII M, MOTOI M, SAEKI H, AOE K AND MORIWAKI S. (1993). Prognostic significance of proliferating cell nuclear antigen (PCNA) expression in non-small cell lung cancer. *Acta Med. Okayama*, **47**, 103–108.

GABBERT HE, WAGNER R AND HÖHN P. (1982). The relation between tumor cell proliferation and vascularization in differentiated and undifferentiated colon carcinomas in the rat. *Virchows Arch. (Cell Pathol.)*, **41**, 119–131.

GABBERT HE. (1985). Mechanisms in tumor invasion: evidence from in vivo observations. *Cancer Metastasis Rev.*, **4**, 293–309.

GABBERT HE, MEIER S, GERHARZ CD AND HOMMEL G. (1991). Incidence and prognostic significance of vascular invasion in 529 gastric cancer patients. *Int. J. Cancer*, **49**, 203–207.

GASPARINI G, BEVILACQUA P, POZZA F, MELI S, BORACCHI P, MARUBINI E AND SAINSBURY JR. (1992*a*). Value of epidermal growth factor receptor status compared with growth fraction and other factors for prognosis in early breast cancer. *Br. J. Cancer*, **66**, 970–976.

GASPARINI G, MELI S, POZZA F, CAZZAVILLAN S AND BEVILACQUA P. (1992*b*). Pc-10 antibody to proliferating cell nuclear antigen (PCNA) is not related to prognosis in human breast carcinoma. *Growth Regul.*, **4**, 145–150.

GERDES J, SCHWAB U, LEMKE H AND STEIN H. (1984). Cell cycle analysis of a cell proliferation-associated human nuclear antigen defined by the monoclonal antibody Ki-67. *J. Immunol.*, **133**, 1710–1715.

HAERSLEV T AND JACOBSEN GK. (1994). Proliferating cell nuclear antigen in breast carcinomas. An immunohistochemical study with correlation to histopathological features and prognostic factors. *Virchows Arch.*, **424**, 39–46.

HARPER ME, GLYNNE-JONES E, GODDARD L, WILSON DW, MATENHELIA SS, CONN IG, PEELING WB AND GRIFFITHS K. (1992). Relationship of proliferating cell nuclear antigen (PCNA) in prostatic carcinomas to various clinical parameters. *Prostate*, **20**, 243–253.

HEMMING AW, DAVIS NL, KLUFTINGER A, ROBINSON B, QUENVILLE NF, LISEMAN B AND LERICHE J. (1992). Prognostic markers of colorectal cancer: an evaluation of DNA content, epidermal growth factor receptor, and Ki-67. *J. Surg. Oncol.*, **51**, 147–152.

HENRIKSEN R, STRANG P, BACKSTROM T, WILANDER E, TRIBUKAIT B AND OBERG K. (1994). Ki-67 immunostaining and DNA flow cytometry as prognostic factors in epithelial ovarian cancers. *Anticancer Res.*, **14**, 603–608.

HERMANEK P, AND SOBIN LH (EDS). (1987). *TNM Classification of Malignant Tumours*. pp. 43–46. Springer: Berlin.

ISOLA J, KALLIONIEMI OP, KORTE JM, WAHLSTROM T, AINE R, HELL M AND HELIN H. (1990). Steroid receptors and Ki-67 reactivity in ovarian cancer and in normal ovary: correlation with DNA flow cytometry, biochemical receptor assay, and patient survival. *J. Pathol.*, **162**, 295–301.

JAIN S, FILIPE MI, HALL PA, WASEEM N, LANE DP AND LEVISON DA. (1991). Prognostic value of proliferating cell nuclear antigen in gastric carcinoma. *J. Clin. Pathol.*, **44**, 655–659.

JORDAN PA, KERNS BJ, PENCE JC, KOHLER MF, BAST RC Jr, KINNEY RB AND BERCHUCK A. (1993). Determination of proliferation index in advanced ovarian cancer using quantitative image analysis. *Am. J. Clin. Pathol.*, **99**, 736–740.

KAKEJI Y, KORENAGA D, TSUJITANI S, HARAGUCHI M, MAEHARA Y AND SUGIMACHI K. (1991). Predictive value of Ki-67 and argyrophilic nucleolar organizer region staining for lymph node metastasis in gastric cancer. *Cancer Res.*, **51**, 3503–3506.

KALBFLEISCH JD AND PRENTICE RL. (1980). *The Statistical Analysis of Failure Data*. Wiley: New York.

KAPLAN EL AND MEIER P. (1958). Nonparametric estimation from incomplete observations. *J. Am. Stat. Assoc.*, **53**, 457–481.

KERNS BM, JORDAN PA, FAERMAN LL, BERCHUCK A, BAST RC AND LAYFIELD LJ. (1994). Determination or proliferation index with MIB-1 in advanced ovarian cancer using quantitative image analysis. *Am. J. Clin. Pathol.*, **101**, 192–197.

KEY G, BECKER MHG, DUCHROW M, SCHLÜTER C AND GERDES J. (1992). New Ki-67 equivalent murine monoclonal antibodies (MIB 1–3) prepared against recombinant parts of the Ki-67 antigen. *Anal. Cell. Pathol.*, **4**, 181–188.

KUBOTA Y, PETRAS RE, EASLEY KA, BAUER TW, TUBBS RR AND FAZIO VW. (1992). Ki-67-determined growth fraction versus standard and grading parameters in colorectal carcinoma. *Cancer*, **70**, 2602–2609.

LAUREN P. (1965). The two histological main types of gastric carcinomas: diffuse and so-called intestinal-type carcinoma. *Acta Pathol. Microbiol. Immunol. Scand.*, **64**, 31-49.

LIPPONEN PK AND ESKELINEN MJ. (1992). Cell proliferation of transitional cell bladder tumours determined by PCNA/cyclin immunostaining and its prognostic value. *Br. J. Cancer*, **66**, 171-176.

MACDONALD-BRAVO H AND BRAVO R. (1985). Induction of the nuclear protein cyclin in serum-stimulated quiescent 3T3 cells is independent of DNA synthesis. *Exp. Cell Res.*, **156**, 455.

MAEDA K, CHUNG YS, TAKATSUKA S, OGAWA Y, ONODA N, SAWADA T, NITTA A, ARIMOTO Y, KONDO Y AND SOWA M. (1995). Tumour angiogenesis and tumour cell proliferation as prognostic indicators in gastric carcinoma. *Br. J. Cancer*, **72**, 319-323.

MAYER A, TAKIMOTO M, FRITZ E, SCHELLANDER G, KOFLER K AND LUDWIG H. (1993). The prognostic significance of proliferating cell nuclear antigen, epidermal growth factor receptor, and mdr gene expression in colorectal cancer. *Cancer*, **71**, 2454-2460.

MORI M, KAKEJI Y, ADACHI Y, MORIGUCHI S, MAEHARA Y, SUGIMACHI K, JESSUP JM, CHEN LB AND STEELE GD Jr. (1993). The prognostic significance of proliferating cell nuclear antigen in clinical gastric cancer. *Surgery*, **113**, 683-690.

MULDER AH, VAN HOOTEGEM JC, SYLVESTER R, TEN KATE FJ, KURTH KH, OOMS EC AND VAN DER KWAST TH. (1992). Prognostic factors in bladder carcinoma: histologic parameters and expression of a cell cycle-related nuclear antigen (Ki-67). *J. Pathol.*, **166**, 37-43.

OOTA K AND SOBIN LH. (1977). *Histological Typing of Gastric and Oesophageal Tumours.* International Histological Classification of Tumours. WHO, Geneva. Springer: Berlin.

QUINN CM AND WRIGHT NA. (1990). The clinical assessment of proliferation and growth in human tumours: evaluation of methods and applications as prognostic variables. *J. Pathol.*, **160**, 93-102.

PORSCHEN R, KRIEGEL A, LANGEN C, CLASSEN S, HILSE M, LOHE B, HENGELS KJ AND BORCHARD F. (1991). Assessment of proliferative activity in carcinomas of the human alimentary tract by Ki-67 immunostaining. *Int. J. Cancer*, **47**, 686-691.

RAILO M, NORDLING S, VON BOGUSLAWSKY K, LEIVONEN M, KYLLONEN L AND VON SMITTEN K. (1993). Prognostic value of Ki-67 immunolabelling in primary operable breast cancer. *Br. J. Cancer*, **68**, 579-583.

SIITONEN SM, KALLIONIEMI OP AND ISOLA JJ. (1993). Proliferating cell nuclear antigen immunohistochemistry using monoclonal antibody 19A2 and a new antigen retrieval technique has prognostic impact in archival paraffin-embedded node-negative breast cancer. *Am. J. Pathol.*, **142**, 1081-1089.

SKOPELITOU A, KORKOLOPOULOU P, PAPANICOLAOU A, CHRISTODOULOU P, THOMAS-TSAGLI E AND PAVLAKIS K. (1992). Comparative assessment of proliferating cell nuclear antigen immunostaining and of nucleolar organizer region staining in transitional cell carcinomas of the urinary bladder. Correlation with other conventional prognostic pathologic parameters. *Eur. Urol.*, **22**, 235-240.

THOMAS H, NASIM MM, SARRAF CE, ALISON MR, LOVE S, LAMBERT HE AND PRICE P. (1995). Proliferating cell nuclear antigen (PCNA) immunostaining-a prognostic factor in ovarian cancer? *Br. J. Cancer*, **71**, 357-362.

THOMAS M, NOGUCHI M, KITAGAWA H, KINOSHITA K AND MIYAZAKI I. (1993). Poor prognostic value of proliferating cell nuclear antigen labelling index in breast carcinoma. *J. Clin. Pathol.*, **46**, 525-528.

TUBIANA M, PEJOVIC MH, KOSCIELNY S, CHVAUDRA N AND MALAISE E. (1989). Growth rate, kinetics of tumor cell proliferation and long-term outcome in human breast cancer. *Int. J. Cancer*, **44**, 17-22.

YONEMURA Y, OHOYAMA S, KIMURA H, KAMATA T, YAMAGUCHI A AND MIYAZAKI I. (1990*a*). Assessment of tumor cell kinetics by monoclonal antibody Ki-67. *Eur. Surg. Res.*, **22**, 365-370.

YONEMURA Y, OHOYAMA S, SUGIYAMA K, NINOMIYA I, KAMATA T, YAMAGUCHI A, MATSUMOTO H AND MIYAZAKI I. (1990*b*) Growth fractions in gastric carcinomas determined with monoclonal antibody Ki-67. *Cancer*, **65**, 1130-1134.

YONEMURA Y, OHOYAMA S, KIMURA T, MATSUMOTO H, YAMAGUCHI A, KOSAKA T, MIWA K AND MIYAZAKI I. (1991). The expression of proliferative-associated nuclear antigen p105 in gastric carcinoma. *Cancer*, **67**, 2523-2528.

YONEMURA Y, KIMURA H, FUSHIDA S, TUGAWA K, NAKAI Y, KAJI M, FONSECA L, YAMAGUCHI A AND MIYAZAKI I. (1993). Analysis of proliferative activity using anti-proliferating cell nuclear antigen antibody in gastric cancer tissue specimens obtained by endoscopic biopsy. *Cancer*, **71**, 2448-2453.